# Heterodonty and ontogenetic shift dynamics in the dentition of the tiger shark *Galeocerdo cuvier* (Chondrichthyes, Galeocerdidae)

**DOI:** 10.1111/joa.13668

**Published:** 2022-04-15

**Authors:** Julia Türtscher, Patrick L. Jambura, Faviel A. López‐Romero, René Kindlimann, Keiichi Sato, Taketeru Tomita, Jürgen Kriwet

**Affiliations:** ^1^ Faculty of Earth Sciences, Geography and Astronomy Department of Palaeontology University of Vienna Vienna Austria; ^2^ Vienna Doctoral School of Ecology and Evolution (VDSEE), University of Vienna Vienna Austria; ^3^ Haimuseum und Sammlung R. Kindlimann Aathal‐Seegräben Switzerland; ^4^ Okinawa Churashima Research Center Okinawa Churashima Foundation Motobu‐cho Okinawa Japan; ^5^ Okinawa Churaumi Aquarium Okinawa Churashima Foundation Motobu‐cho Okinawa Japan

**Keywords:** elasmobranch, embryonic dentition, geometric morphometrics, ontogenetic trajectory, teeth, tooth pattern reversal

## Abstract

The lifelong tooth replacement in elasmobranch fishes (sharks, rays and skates) has led to the assemblage of a great number of teeth from fossil and extant species, rendering tooth morphology an important character for taxonomic descriptions, analysing phylogenetic interrelationships and deciphering their evolutionary history (e.g. origination, divergence, extinction). Heterodonty (exhibition of different tooth morphologies) occurs in most elasmobranch species and has proven to be one of the main challenges for these analyses. Although numerous shark species are discovered and described every year, detailed descriptions of tooth morphologies and heterodonty patterns are lacking or are only insufficiently known for most species. Here, we use landmark‐based 2D geometric morphometrics on teeth of the tiger shark *Galeocerdo cuvier* to analyse and describe dental heterodonties among four different ontogenetic stages ranging from embryo to adult. Our results reveal rather gradual and subtle ontogenetic shape changes, mostly characterized by increasing size and complexity of the teeth. We furthermore provide the first comprehensive description of embryonic dental morphologies in tiger sharks. Also, tooth shapes of tiger sharks in different ontogenetic stages are re‐assessed and depicted in detail. Finally, multiple cases of tooth file reversal are described. This study, therefore, contributes to our knowledge of dental traits across ontogeny in the extant tiger shark *G. cuvier* and provides a baseline for further morphological and genetic studies on the dental variation in sharks. Therefore, it has the potential to assist elucidating the underlying developmental and evolutionary processes behind the vast dental diversity observed in elasmobranch fishes today and in deep time.

## Abstract 2

Using 2D geometric morphometrics, we examined the tooth morphology and heterodonty patterns across ontogeny in extant tiger sharks. Examining tiger sharks in different ontogenetic stages allowed us to provide detailed descriptions of intraspecific tooth variations and to confirm a weak ontogenetic heterodonty in this species.

## 1 INTRODUCTION

During their long evolutionary history, elasmobranchs (sharks, rays and skates) have developed a plethora of morphological traits that allowed them to occupy different ecological niches. Among the key features that facilitated the success of this group certainly is their polyphyodont dentition (teeth are constantly formed and shed throughout an individual's life), which allows them greater morphogenetic plasticity, rapid developmental changes and the formation of a vast diversity of different tooth shapes (Rasch et al., 2016). The development of different dental shapes is often thought to correspond to different feeding strategies (Bazzi et al., 2021; Cappetta, 2012; Frazzetta, 1988; Huber et al., 2009), although evidence for such a link between tooth morphology and function is cloudy at best (Corn et al., 2016; Whitenack & Motta, 2010). Due to this diversity and the circumstance that teeth are the predominant remains of this group in the fossil record, teeth are critical for taxonomic identifications (e.g. Cappetta, 2012; Guinot et al., 2013; Jambura et al., 2021) and inference of phylogenetic relationships between extinct and extant taxa (Gates et al., 2019; Klug, 2010; Landemaine et al., 2018). In extant elasmobranchs, conversely, tooth morphologies are rarely used in species descriptions (Guinot et al., 2018), and therefore, morphological details sufficient for taxonomic differentiation are often missing. However, a thorough knowledge of tooth morphologies is crucial since the fossil record of elasmobranchs consists predominantly of isolated teeth. Establishing tooth morphologies consequently has direct consequences on our understanding of elasmobranch taxonomic diversities in deep time.

An issue that arises when erecting fossil elasmobranch species based on isolated teeth is the fact that not all elasmobranchs exhibit a homodont dentition (i.e. all teeth within an individual have the same tooth shape), but many species exhibit a form of heterodonty (i.e. development of different tooth morphologies within an individual or species; Compagno, 1970). Different tooth shapes can be developed in teeth occupying different tooth positions along the jaw ramus (from mesial to distal; monognathic heterodonty), but also between teeth of the lower and upper jaws (dignathic heterodonty). These morphological changes can be either rather subtle or well pronounced as in bullhead sharks (Heterodontiformes), which have small, multicuspid mesial teeth, but large molariform distal teeth (Herman et al., 1993; Jambura et al., 2020; Reif, 1976). The very distinct monognathic heterodonty in heterodontiforms is thought to be an adaptation to the different functions the teeth have to perform: the small, cuspidate mesial teeth allow heterodontiform sharks to grasp soft‐bodied prey or detach prey from the substrate, while the robust and flattened distal teeth allow them to crack hard‐shelled prey, making them versatile feeders (Edmonds et al., 2001).

Gynandric heterodonty (sexual dimorphism in teeth) is another widespread phenomenon in elasmobranchs that can either be exhibited permanently, or only temporarily during the mating season (Berio et al., 2020; Kajiura & Tricas, 1996; Underwood et al., 2015). In Atlantic stingrays *Hypanus sabinus* (Lesueur, 1824), for example males develop more cuspidate teeth during the mating season, supposedly to get a better grip on the female during mating (Kajiura & Tricas, 1996). Temporal tooth variations are facilitated by the polyphyodont tooth replacement and are not restricted to gynandric heterodonty, but can also occur throughout ontogeny dependent or independent of sex (Berio et al., 2020; French et al., 2017; Herman et al., 1993; Hubbell, 1996; Purdy & Francis, 2007). Such ontogenetic shifts in tooth morphologies are often linked to dietary shifts, for example in large predatory species such as the white shark: juvenile white sharks are predominantly piscivorous as they have not yet reached the body size to hunt and feed on large prey, while older specimens mainly feed on marine mammals (Cliff et al., 1989; Estrada et al., 2006; Hussey et al., 2012; Tricas & McCosker, 1984). Additionally, dietary shifts might facilitate resource partitioning and thus decrease intraspecific competition (Powter et al., 2010). It should be noted that ontogenetic shifts in tooth morphology are seen in some species even before birth; embryonic white sharks *Carcharodon carcharias* (Linnaeus, 1758) were reported to have a distinct tooth morphology as early embryos differing from that of late embryos and neonates which is most likely associated to the oophagous (egg‐eating) phase (Tomita et al., 2017). This reproductive mode, known as lamniform oophagy, is one of several reproductive modes observed in sharks. However, because of the limited data available on embryonic dentitions, it is not known to what extent dental morphology might reflect the reproductive mode. To identify and describe these relationships between reproduction modes and the embryonic dentition it is crucial to gain a more complete picture of ontogenetic heterodonty in sharks in general.

Another large, coastal‐pleagic shark, the tiger shark *Galeocerdo cuvier* (Péron & Lesueur, 1822), has also evolved a distinct but unique reproduction mode termed embryotrophy (Castro et al., 2016): during development, tiger shark embryos are surrounded by a thin diaphanous egg case, which is filled with a yellowish fluid (Castro et al., 2016) from which they emerge during parturition (Tomita et al., 2018). Besides being nourished by the yolk sac, tiger shark embryos additionally imbibe the nutritive fluid (i.e. embryotrophe) inside the egg case (Castro et al., 2016). However, little is known about the embryonic development and the shaping and timing of different morphological structures including teeth in embryonic tiger sharks and it therefore remains elusive whether tiger shark embryos develop distinct tooth morphologies related to intrauterine feeding similar to the condition seen in the white shark.

In contrast, the dentition of tiger sharks across other ontogenetic stages has been more extensively studied (e.g. Compagno, 1988; Ebersole et al., 2019; Jambura et al., 2018; Moyer & Bemis, 2017; Türtscher et al., 2021). Qualitative studies found that the teeth of juvenile tiger sharks are narrower, longer and have fewer and less complex serrations than the teeth of older specimens (Compagno, 1988; Ebersole et al., 2019). Adult tiger sharks possess heavily calcified, broad jaws that yield double serrated, cockscomb‐shaped teeth, typifying the cutting tooth morphology (Cappetta, 2012). In combination with the lateral side‐to‐side movement of the head and body (saw‐biting technique sensu Clua et al., 2013), these teeth allow them to even cut through hard tissues like the bony carapace of large sea turtles (Randall, 1992). In combination with their tremendous size of up to 5.5 m (total length) (Holmes et al., 2012) and their broad jaws, their specialized teeth allow tiger sharks to prey on a wide variety of different prey items, including cephalopods, teleosts, marine reptiles, sea birds and other elasmobranchs, but also indigestible anthropogenic objects such as cans, plastic bags, small barrels, pieces of metal, etc. (Compagno, 1984; Gudger, 1949; Randall, 1992).

Even though tiger sharks are a popular research target in general, not much is known about dental ontogenetic shift dynamics in this species, especially during its early ontogenetic stages. However, extended data on ontogenetic heterodonty in this species will not only enhance our understanding of various life‐history traits such as reproduction, form‐function relationships and niche partitioning; this knowledge is also of tremendous importance for understanding fossil faunas and for correctly identifying extinct species, especially those based only on isolated teeth. Therefore, the aim of this study was to gain better insights into the extent of heterodonty and ontogenetic shift dynamics in the tiger shark. Here, we examine and compare the tooth morphology and development in modern tiger sharks of various ontogenetic stages (late embryo, juveniles, subadults and adults) both qualitatively and quantitatively. We used micro‐CT‐scanning and 2D landmark‐based geometric morphometrics to analyse the degree of mono‐ and dignathic heterodonty and the ontogenetic trajectories of tooth shapes across ontogeny in the tiger shark *Galeocerdo cuvier*.

## 2 MATERIAL AND METHODS

### 2.1 Data generation

A total of 21 dried jaws of the tiger shark *Galeocerdo cuvier* were used in the present study (Table S1). The investigated teeth were not removed from the jaws but studied in situ, therefore, only teeth with fully visible tooth crowns were selected. Teeth from all tooth files along both left and right upper and lower jaws were used, except those with severe damage. Most of the teeth were from the first row, however, several teeth from rows two and three were included as well (when sufficiently mineralized), resulting in a total sample size of 779 teeth for analyses. All teeth were photographed labially because of the flattened shape of the labial side that is ideal for 2D geometric morphometric analyses. A digital camera was positioned orthogonally to each individual tooth to prevent doubtful results due to an erroneous angle. The generated images were rotated using Adobe Photoshop CS6 (version 13.0, Adobe Systems) so that the cusps of all teeth were oriented to the right.

### 2.2 Determination of size and ontogenetic stage

The total lengths of the studied specimens were estimated based on the method proposed by Lowry et al. (2009), who regressed different measurements of the jaw against the known total length (TL) of 14 different shark species, including the tiger shark. Hereby these authors developed an algorithm to calculate an estimated total length on the basis of selected jaw measurements (i.e. interdental distances and bite circumference), which can be applied on fresh and dried jaws as well as on bite damages.

We measured the bite circumference (BC) of both the lower and upper jaws, which is defined as the distance between the two distalmost teeth of the functional tooth row along the consecutive tooth bases. The measurements were imported into the spreadsheet provided by Lowry et al. (2009), which resulted in two values for the estimated total length (computed through the BC of the palatoquadrate cartilage and the Meckel's cartilage, respectively). Unsurprisingly, both values barely differed from each other (see Table S1), and the arithmetic mean of both values was used to estimate the ontogenetic stage for each specimen.

**FIGURE 1:**
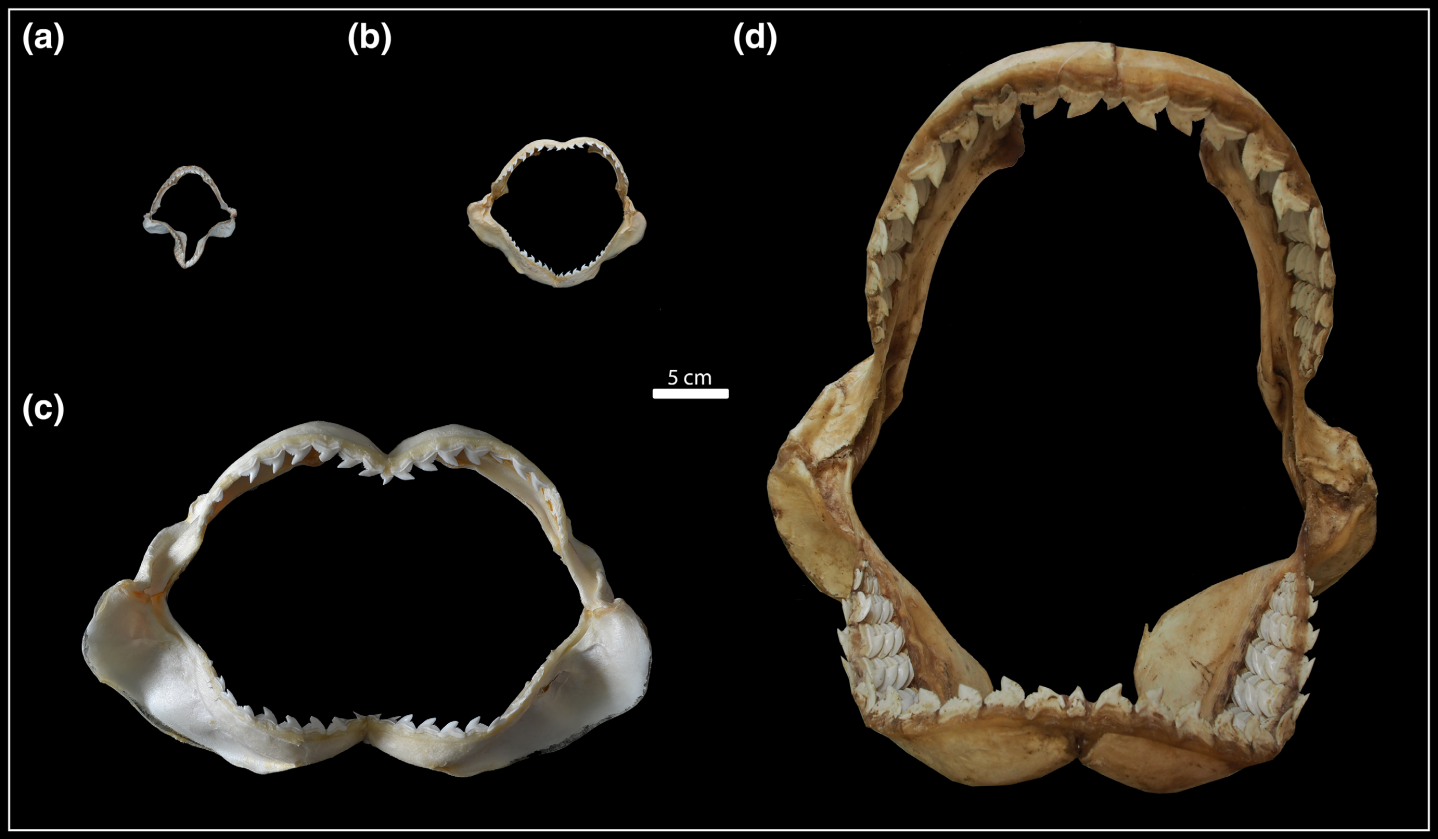
Four tiger shark jaws to exemplify the ontogenetic stages studied. (a) embryo (EMRG‐Chond‐A‐1b) ‐ (b) juvenile (EMRG‐Chond‐J‐11) – (c) subadult (EMRG‐Chond‐J‐9) – (d) adult (HNS‐Pisc‐S‐0019)

Based on previous studies on the growth and ontogeny of the tiger shark (Branstetter et al., 1987; Stevens & McLoughlin, 1991; Simpfendorfer, 1992; Heithaus, 2001; Ebert et al., 2013; Holmes et al., 2015), we distinguished between the following ontogenetic categories: embryo: 0–79 cm TL, juvenile: 80–199 cm TL, subadult: 200–299 cm TL and adult: 300–550 cm TL (Figure 1). Accordingly, we had one embryo, eight juvenile, seven subadult and five adult specimens in our study (Table S1).

### 2.3 Dental terminology

**FIGURE 2:**
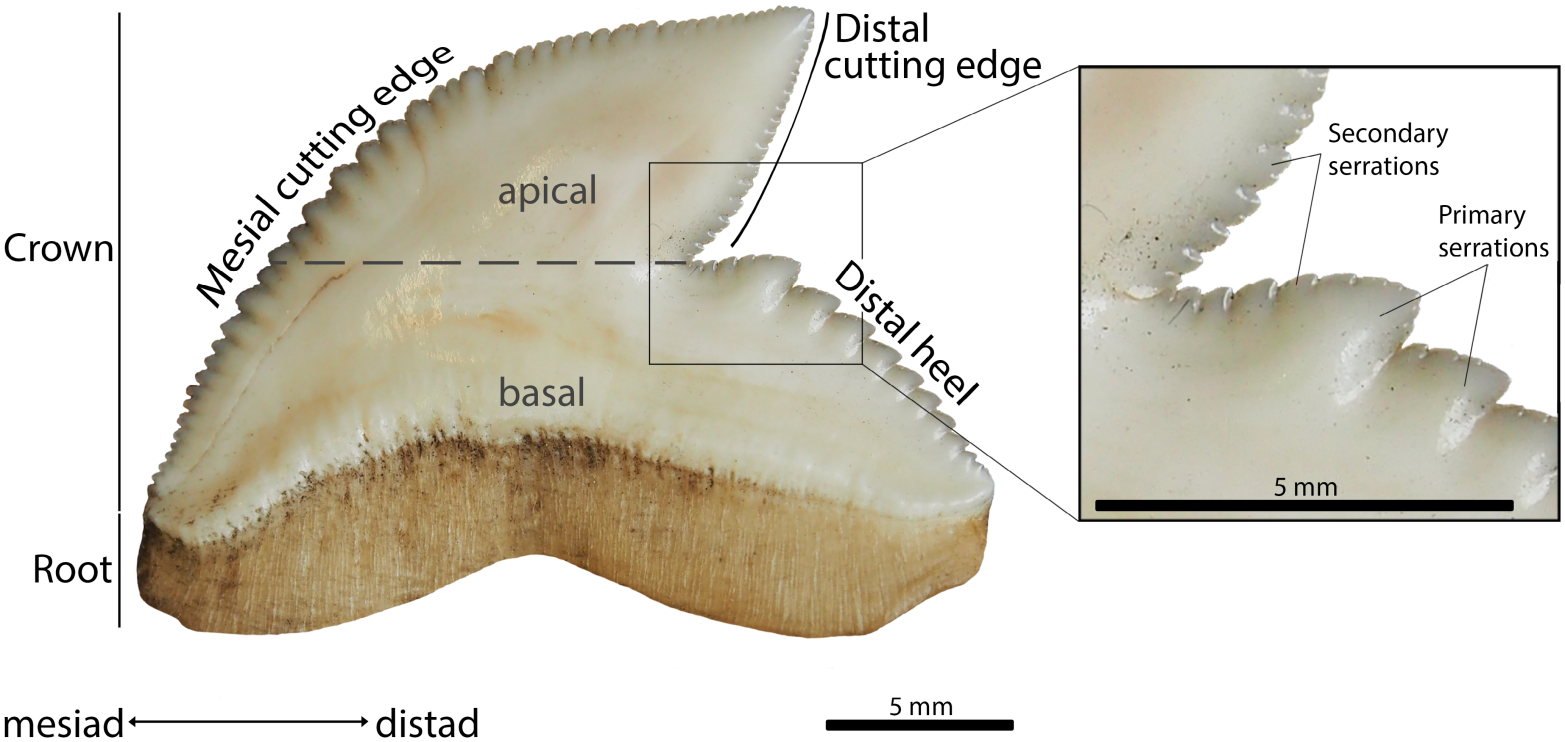
Terminology used to describe the individual teeth. Photograph shows the labial side of a tooth of an adult *G. cuvier* specimen (EMRG‐Chond‐J‐16, first replacement tooth of RP6). Modified after Türtscher et al. (2021)

The terminology used for the individual teeth follows Türtscher et al. (2021). Teeth of *Galeocerdo cuvier* are asymmetrical as the crown is compressed and distally inclined. The mesial cutting edge is curved, whereas the distal edge is deeply notched and divided into the distal heel and distal cutting edge. The crown is completely serrated with compound serrations, whereby large primary serrations are located on the mesial cutting edge and the distal heel, while secondary serrations are situated on and between primary serrations as well as on the distal cutting edge (Moyer & Bemis, 2017) (Figure 2).

Teeth were regarded as functional when fully mineralized and in an erect position (following Moyer et al. (2015), Schnetz et al. (2016) and Jambura et al. (2018)).

The terminology used for tooth files is modified after Moyer et al. (2015): the first letter is optional and describes the side of the jaw (L—left, R—right). The next letter refers to the upper (P—palatoquadrate) or lower jaw (M—Meckel's cartilage). This is followed by the exact position of the tooth file (S—symphyseal tooth file, 1—first tooth file next to the symphysis, etc.). Accordingly, for example the eight tooth file in the left palatoquadrate is LP8.

### 2.4 CT‐Scanning and 3D‐modelling

Tooth development, tooth count within tooth files and tooth histology were investigated qualitatively using two different micro‐CT devices: a SkyScan1173 micro‐CT device (Bruker/Skyscan, Kontich, Belgium) at the Department of Palaeontology, University of Vienna (Austria), and a Viscom X8060 NDT X‐ray (Viscom AG, Hannover, Germany) at the Department of Anthropology, University of Vienna (Austria).

A whole jaw for each ontogenetic stage, that is embryonic (EMRG‐Chond‐A‐1b; voxel size 19.63 μm for the upper jaw, 20.66 μm for the lower jaw), juvenile (EMRG‐Chond‐J‐13; voxel size 35.62 μm), subadult (EMRG‐Chond‐J‐10; voxel size 80 μm) and adult tiger shark (EMRG‐Chond‐J‐16; voxel size 75 μm) was scanned using micro‐computed tomography. Additionally, a single tooth was removed and scanned from the embryo (EMRG‐Chond‐A‐1d; voxel size 5.00 μm) and a juvenile specimen (EMRG‐Chond‐T‐79; voxel size 5.00 μm) and then compared to a tooth of an adult specimen (EMRG‐Chond‐T‐16; voxel size 14.96 μm) to examine the histology of the serrations in each of these stages. Three‐dimensional volume‐rendered reconstructions of the scanned jaws and teeth were created by loading the resulting stack files into Amira (version 5.4.5, FEI Visualization Sciences Group, Oregon, USA). This software package further allowed us to virtually section the teeth at every favoured position to investigate the tooth development and the number of teeth per tooth file. The resulting 2D images were edited in terms of colour balance, contrast and labelling using the program Adobe Photoshop CS6.

For each ontogenetic stage, a tooth row of the upper and lower jaw was reconstructed three‐dimensionally using Amira and the online platform Biomedisa (Lösel et al., 2020).

### 2.5 Geometric morphometrics

**FIGURE 3:**
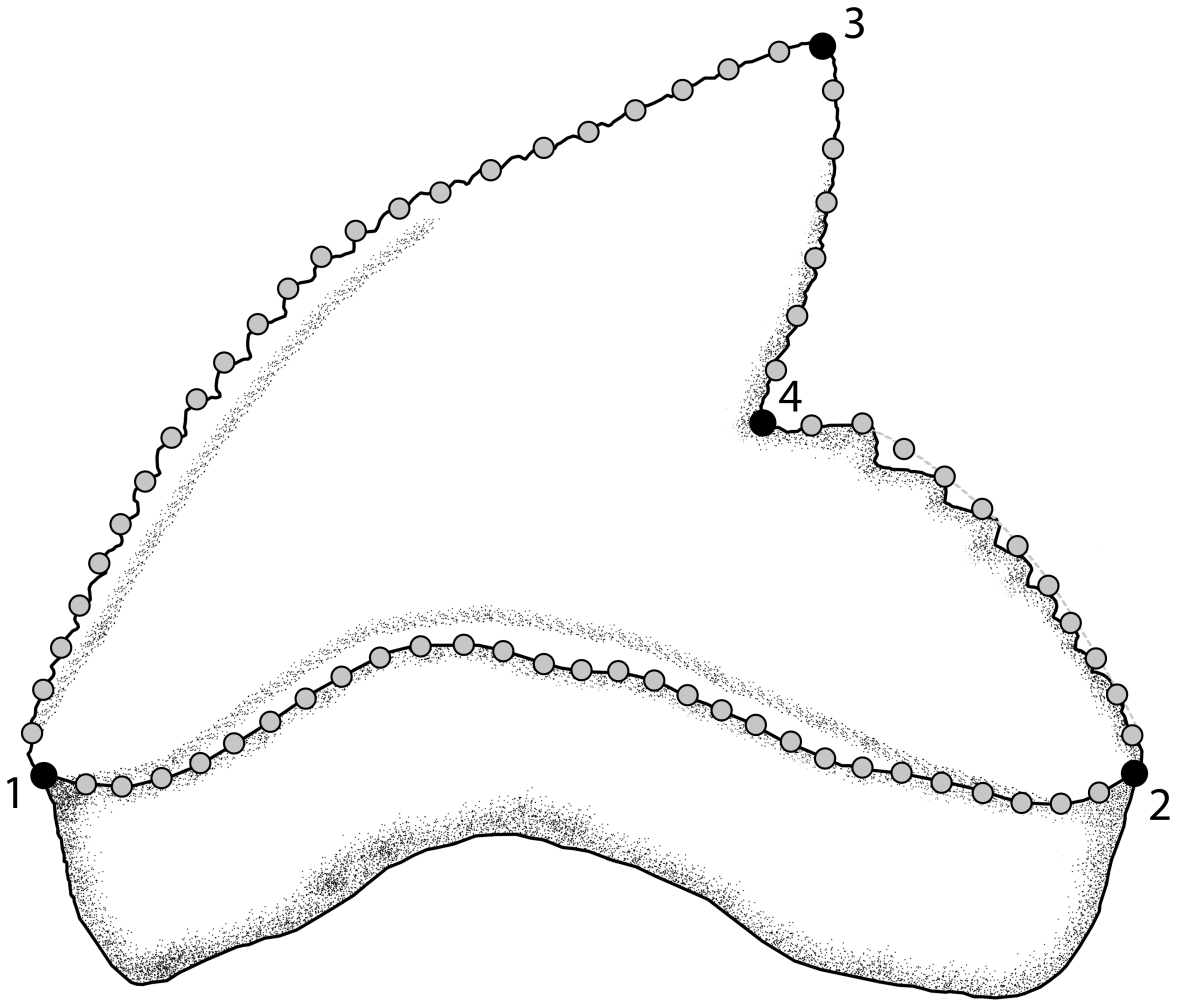
Location of the landmarks and semilandmarks for the geometric morphometric analyses. The landmarks are located on the (1) base of mesial cutting edge, (2) base of distal cutting edge, (3) tip of cusp and (4) distal notch. Twenty‐three semilandmarks are located between the base of the mesial cutting edge and the tip of the cusp, six are located between the tip of the cusp and the distal notch, 11 semilandmarks are situated between the distal notch and the base of the distal cutting edge, and 28 are located along the crown‐root boundary between the base of the distal and mesial cutting edge

The tooth shape of *Galeocerdo cuvier* was studied using 2D landmark‐based geometric morphometrics. Four homologous landmarks were digitized using the software tpsDIG2 (v. 2.31; Rohlf, 2017). Additionally, 68 semilandmarks were digitized between the homologous landmarks to capture the overall tooth shape (Figure 3). To minimize the variance caused by size, orientation, location and rotation, a generalized Procrustes analysis (GPA) was performed on the landmark coordinates (Rohlf & Slice, 1990). The sliding semilandmarks were allowed to slide to minimize the bending energy (Bookstein, 1997). The aligned coordinates were then subjected to a principal component analysis (PCA) to assess shape variation of teeth. An analysis of similarities (ANOSIM; Clarke, 1993) was implemented to evaluate the extent of overlap between the generated morphospaces of teeth from upper and lower jaws. Tooth shape differences within the different ontogenetic stages of *Galeocerdo cuvier* were estimated with a permutational analysis of variance (ANOVA) with 1000 permutations, followed by pairwise comparisons between the groups (centroid size, ontogenetic stage, jaw side [left/right], palatoquadrate/Meckel's cartilage, tooth position within the mesio‐distal axis of the jaws), with the functions *procD.lm* and *pairwise* considering the distances between means in the R packages *geomorph* (v. 3.1; Adams et al., 2016) and *RRPP* (Collyer & Adams, 2018).

To assess the ontogenetic shape change within each tooth position as well as the shape change of teeth along the mesio‐distal axis for each ontogenetic group we performed trajectory analyses using the function *trajectory. analysis* in the R package *RRPP* (Collyer & Adams, 2018).

## 3 RESULTS

### 3.1 Tooth file arrangement

All examined jaws exhibit one file of symphyseal teeth on both the palatoquadrate and Meckel's cartilage. The amount of tooth files on the palatoquadrate and Meckel's cartilage of all ontogenetic groups ranges from 10 (*n* = 9) to 12 (*n* = 14) tooth files for each side (left and right jaw rami). The most frequent number of tooth files observed is 11 (*n* = 60), resulting in a median number of 11 tooth files. The average tooth file number, however, is slightly higher for the Meckel's cartilage than for the palatoquadrate (Table S1).

**FIGURE 4:**
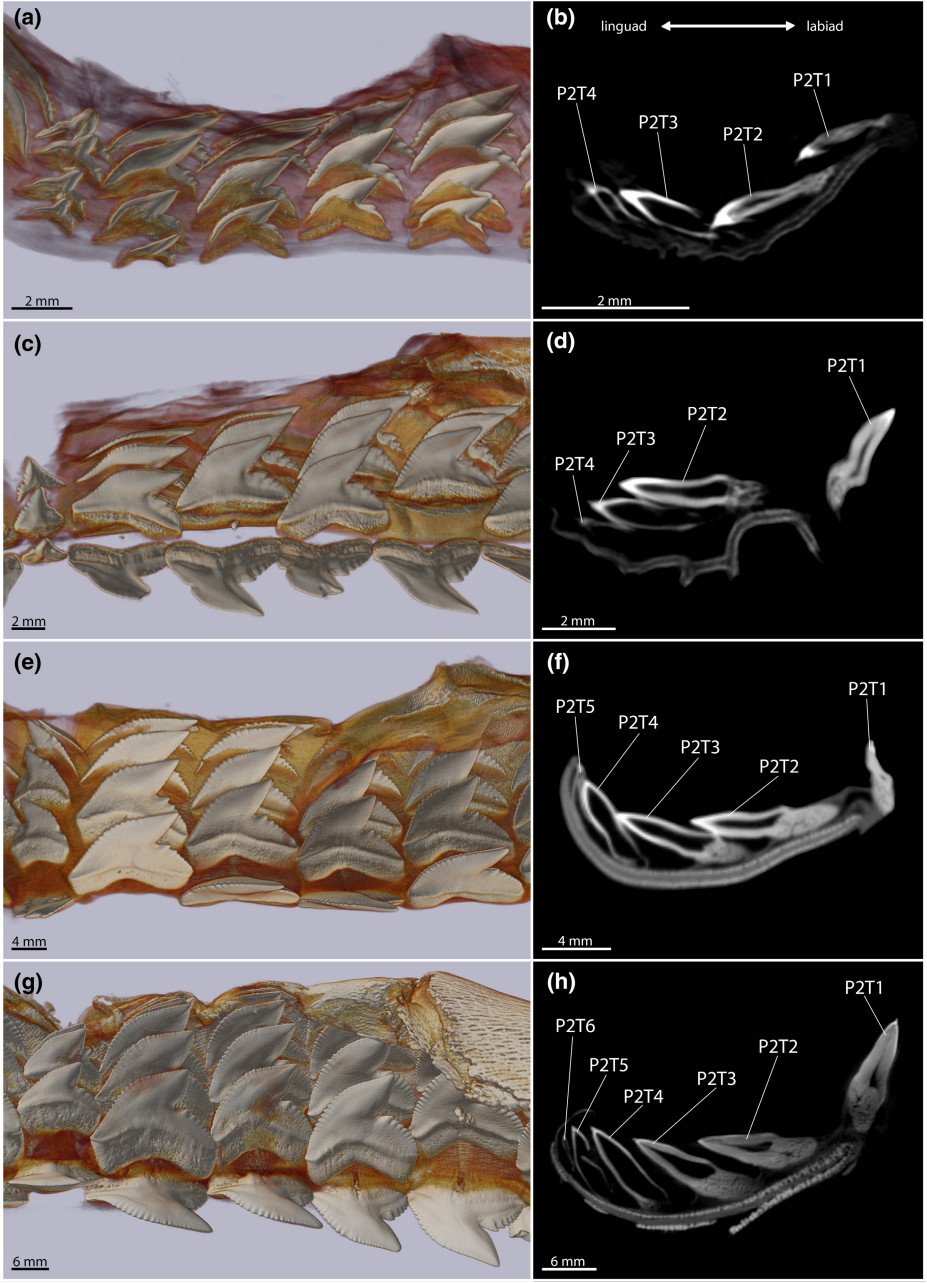
Three‐dimensional reconstructions of four tiger shark palatoquadrates visualizing the tooth morphology through ontogeny and virtual sections through tooth files; embryo (EMRG‐Chond‐A‐1b) – (a) tooth files LPS‐4 – (b) sectioned tooth file LP2; juvenile (EMRG‐Chond‐J‐13) – (c) tooth files RPS‐4 – (d) sectioned tooth file RP2; subadult (EMRG‐Chond‐J‐10) – (e) tooth files RPS‐4 – (f) sectioned tooth file RP2; adult (EMRG‐Chond‐J‐16) – (g) tooth files RPS‐4 – (h) sectioned tooth file RP2. P2, second tooth file next to the symphysis in the palatoquadrate; T1‐6, teeth one to six within the tooth file, labio‐lingual direction

The number of teeth per tooth file increases during ontogeny, from four teeth per file in the embryonic and juvenile specimens to up to seven teeth per file in adults (Figure 4; Table S2). The oldest, labial‐most tooth is mostly in a functional position, whereas the remaining teeth are regarded as replacement teeth in all ontogenetic groups except the embryonic stage. No functional teeth are present in the embryo, in which all teeth are in a lingually directed replacement position. The labial‐most teeth in the tooth files are generally smaller than the successive replacement teeth. This size discrepancy between functional and replacement teeth is especially apparent in the embryonic specimen and becomes more and more subtle with increased size and age of the specimens. The number of serrae on the distal heel increases in relation to the overall size enlargement.

**FIGURE 5:**
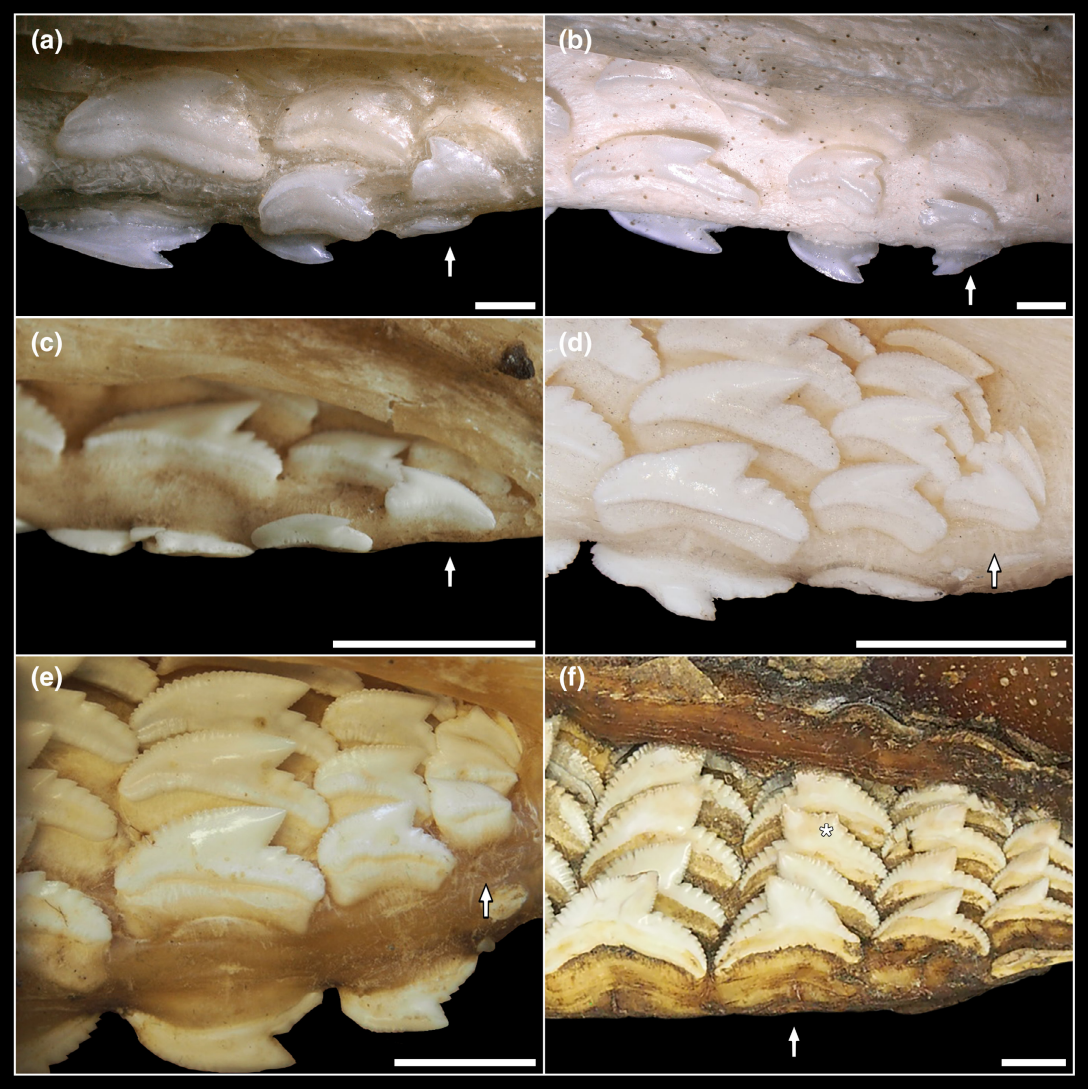
Reversed tooth files, indicated by arrows. (a) juvenile (EMRG‐Chond‐J‐14), LM12 – (b) juvenile (7‐729/RZ), RM11 – (c) subadult (EMRG‐Chond‐J‐22), LM11 – (d) subadult (7‐731/RZ), LM12 – (e) adult (HNS‐Pisc‐S‐0019), LM12 – (f) adult (NHMW‐2001444), LP8. The asterisk indicates enlarged serrations on the mesial cutting edge. Scale bars (a, b) = 1 mm, (c–f) = 10 mm

Of the 21 examined jaws, six (28.57%) exhibit a dental aberration in the form of a whole tooth file with reversed polarity, that is the apex is oriented mesially instead of distally (Figure 5). Five of the affected files (83.3%) are distal‐most tooth files on the Meckel's cartilage, only one tooth file (16.6%) is located further mesial (file eight) and on the left palatoquadrate. The third tooth of this reversed file additionally shows a slight aberration, with unusually enlarged serrations on the mesial cutting edge (Figure 5f). No identifiable underlying causes of the reversed polarity or the malformation of the tooth crown, such as recognizable damages on the jaws and stingray or teleost spines embedded in the jaws (see e.g. Andre, 1784; Becker et al., 2000; Gudger, 1937) are present.

### 3.2 Tooth morphology and arrangement across ontogeny

**FIGURE 6:**
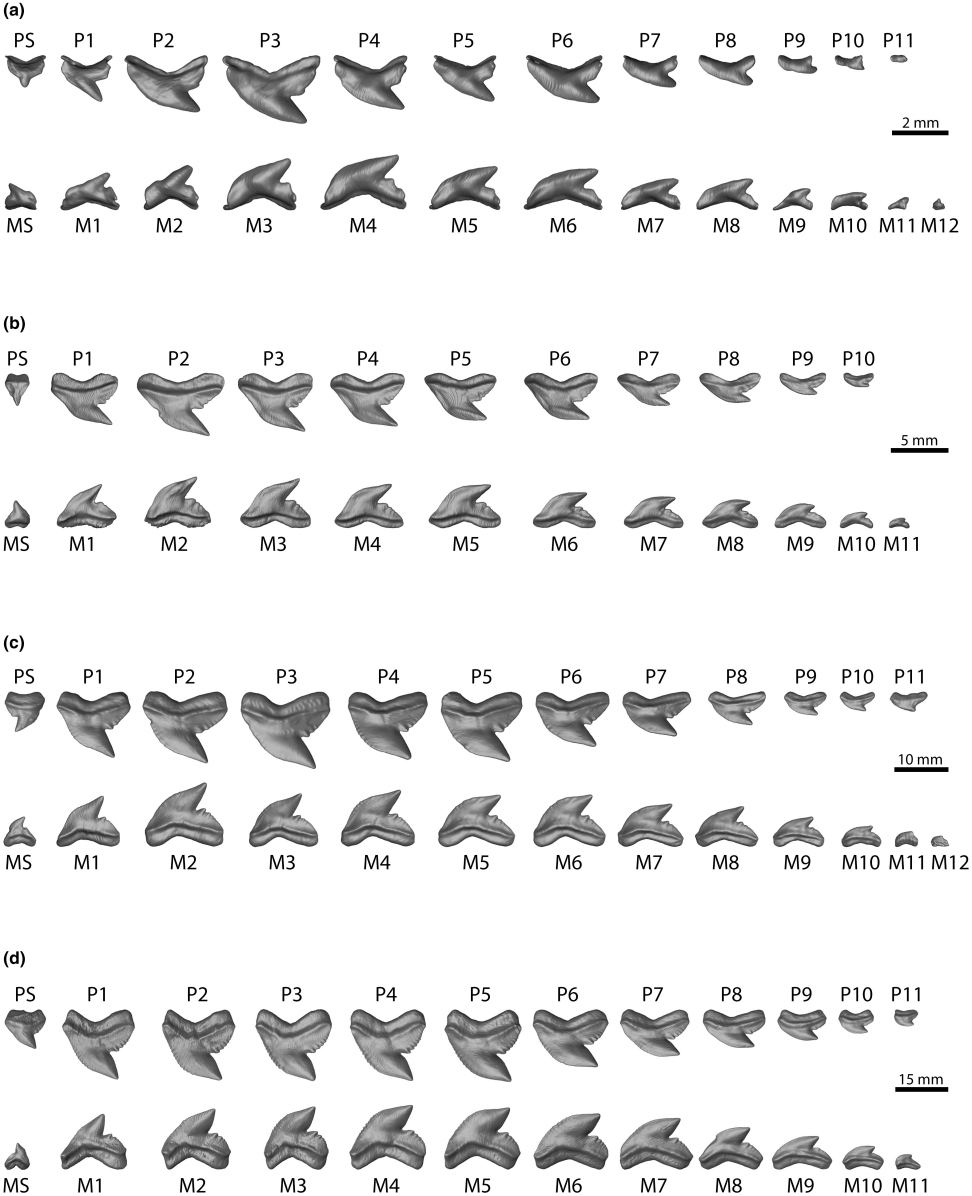
Three‐dimensional reconstruction of the labial‐most tooth row of the palatoquadrate and Meckel's cartilage of four tiger shark specimens. (a) embryo (EMRG‐Chond‐A‐1b) – (b) juvenile (EMRG‐Chond‐J‐13) – (c) subadult (EMRG‐Chond‐J‐10) – (d) adult (EMRG‐Chond‐J‐16)

**FIGURE 7:**
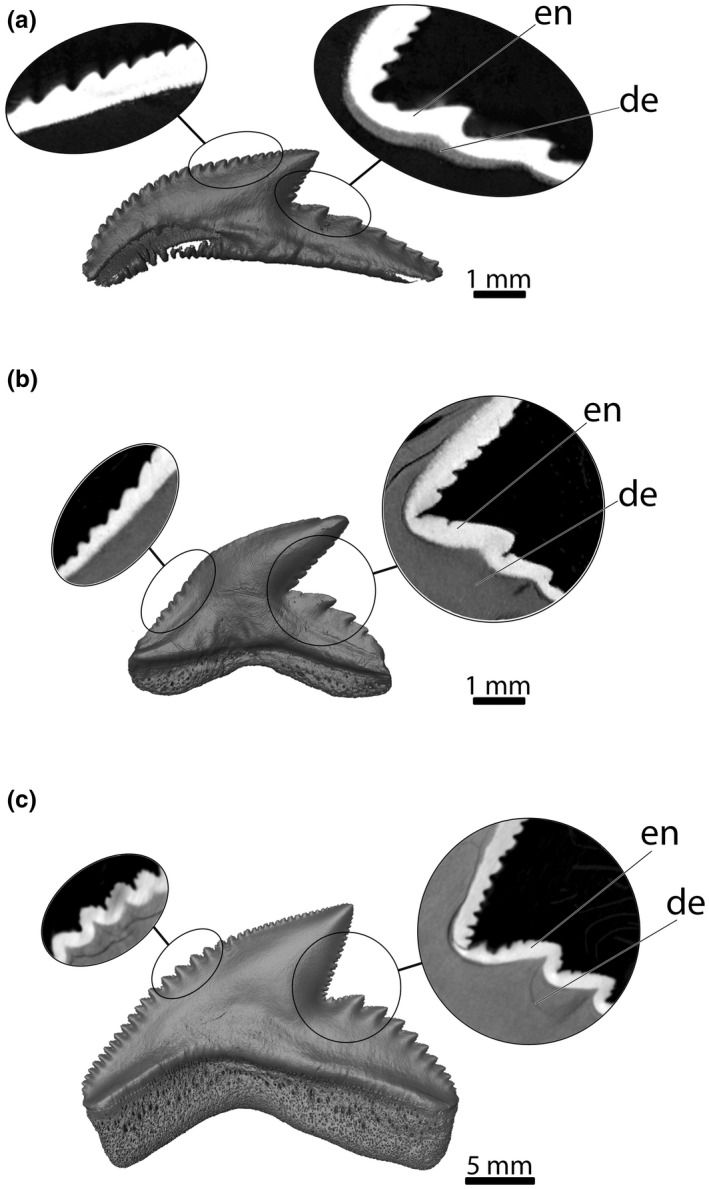
Three‐dimensional reconstructions and virtual sections through single teeth. (a) embryo (EMRG‐Chond‐A‐1d), lingual tooth exhibiting the juvenile tooth morphology – (b) juvenile (EMRG‐Chond‐T‐79), functional tooth – (c) adult (EMRG‐Chond‐T‐16), functional tooth. de, dentine; en, enameloid

The morphological changes from embryonic to adult specimens occur gradually rather than abruptly (Figure 6). The oldest (labial‐most) teeth of the embryo are small and narrow‐crowned and still in a replacement position. They either possess no serrations at all or only subtle traces of serrations. Teeth positioned more lingually are noticeably larger and are serrated with simple serrations. Moyer and Bemis (2017) investigated the tooth histology of an adult tiger shark and proposed that primary serrations are composed by enameloid and dentine, while secondary serrations consist of enameloid only. In the present study, we examined the histology of tooth serrations in three different ontogenetic stages (Figure 7). The serrations seen on the lingually positioned teeth in the embryonic tiger shark specimen are putatively primary serrations based on external morphology, however, histology only confirms that the serrations on the distal heel are true primary serrations as they are already intruded by the developing dentine. In contrast, the serrations on the distal and mesial cutting edges are composed entirely of enameloid (Figure 7a).

**FIGURE 8:**
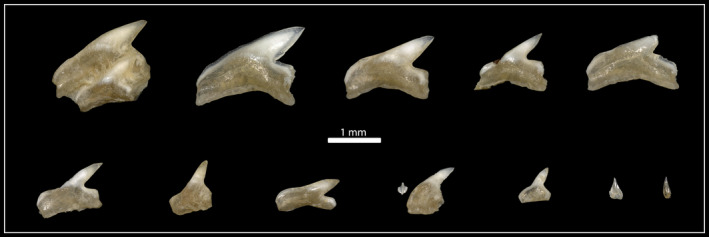
Shed and swallowed teeth, recovered from the stomach of the embryonic specimen (EMRG‐Chond‐A‐1b)

Teeth of the second row (lingual from the labial‐most positioned teeth) are characterized by a serrated distal heel and traces of a serration on the mesial cutting edge, teeth of row three and four are nearly fully serrated, only the apex of the crown is smooth. The serrations are most pronounced in mesial tooth files and decrease in distal tooth files. The increased size of the more lingually positioned teeth lessens the gap spacing between the tooth files and the teeth start to slightly overlap (mixed alternate and imbricate overlap sensu Strasburg (1963)) in the third and fourth rows. All teeth are incompletely mineralized, albeit the tooth crowns are generally more mineralized than the roots. The sparsely developed roots are narrow and symmetrical (Figure 4a). We furthermore recovered 13 shed and swallowed teeth from the stomach and intestine of the embryonic specimen (Figure 8). These teeth possess no serrations and exhibit a similar morphology as the labial‐most positioned teeth in the jaws of the specimen.

Teeth of juveniles exhibit the same morphology in all positions from labial to lingual. The mesial cutting edge is often sigmoidal. The root is symmetrical and broader than that in embryonic teeth. The cusp is narrow and the distal cutting edge is straight. Labial‐most teeth are in a functional position and are fully mineralized. All teeth display primary serrations and a moderate secondary serration also is discernible. Histologically, only the serrations on the distal heel are true primary serrations, as they are composed of dentine and enameloid. The serrations on the mesial cutting edge, conversely, consist only of enameloid (Figure 7b).

The spacing between the subsequent teeth within a tooth file is large. The functional teeth are mostly independent and occasionally arranged in a mixed pattern (mixed alternate and imbricate overlapping). The overlap between the replacement teeth is small, however, they are also overlapping in a mixed alternate and imbricate pattern. An increase in size from labial (functional) to lingual (replacement) teeth is noticeable (Figure 4b).

All teeth of subadults exhibit an asymmetrical root and an evenly convex mesial but a straight distal cutting edge. They exhibit primary and secondary serrations. The increase in size from teeth in labial to lingual positions (from functional to replacement teeth) is weak but visible. The spacing between the subsequent teeth is reduced. The functional and replacement teeth are overlapping in a mixed alternate and imbricate pattern (Figure 4c).

Teeth with an asymmetrical and broad root characterize adult dentitions. The mesial and distal cutting edges are convex resulting in a broad cusp. All teeth are fully serrated with distinct primary and secondary serrations, which are also distinguishable histologically (Figure 7c). The teeth are arranged closely to each other and are overlapping (mixed alternate and imbricate overlap). Within our sample of adult specimens, a weak increase in size of teeth from functional teeth in labial positions to replacement teeth in lingual positions is still detectable (Figure 4d).

Two main morphologies of symphyseal teeth are discernible; the first morphotype resembles the typical tiger shark tooth morphology, but it is noticeably smaller compared to the adjacent teeth. The second morphotype is much narrower, mostly upright and close to symmetrical. Both morphotypes were found in either half of the jaw within all ontogenetic groups of our sample (Table S1).

### 3.3 Principal component analysis

The PCA resulted in 144 axes, with the first three accounting for 81.7% of the total variation. All other axes each account for <5% of the total variation. PC1 (54.9%) describes the morphological variation from mesial to distal tooth files, with positive values indicating almost vertical teeth with an asymmetrically arched crown‐root boundary, a broad cusp and a steep distal cutting edge and distal heel, typical for mesially positioned teeth. Negative scores are related to broad and compressed teeth characteristic for distal positions, with a strongly distally inclined cusp creating a deep notch and a short distal cutting edge.

**FIGURE 9:**
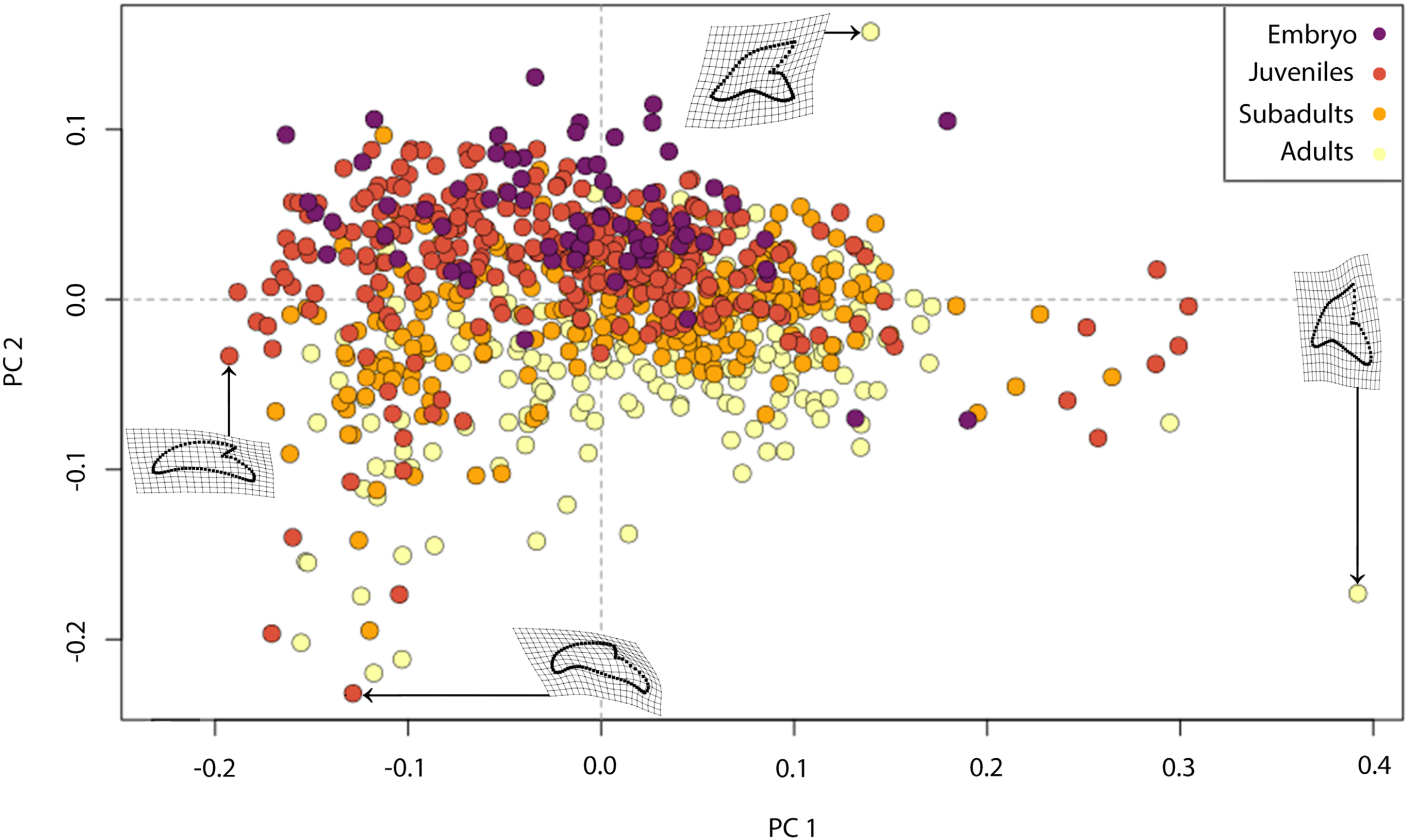
Morphospace occupation of teeth of embryonic, juvenile, subadult and adult tiger sharks. Deformation grids visualize the extreme shapes that lie along the extreme values of the axes. Morphospace plotted on PC1 (54.9%) and PC2 (17.42%)

Positive values of PC2 (17.42%) are related to teeth with a slender cusp, a slightly angular mesial cutting edge, strongly notched distal edges and a symmetrically arched crown‐root boundary. Negative values indicate mesio‐distally compressed teeth with a rather obtuse‐angled and squat cusp, a short distal cutting edge, an elongated and slanted distal heel and an asymmetrical crown‐root boundary. PC2 mainly describes the differences between ontogenetic stages, with embryonic and juvenile specimens mainly accumulating in the positive area. Subadult specimens are distributed equally in the positive and negative areas, while adults are mostly accumulated in the negative realm (Figure 9).

A Procrustes ANOVA revealed significant differences in centroid size (*R* ^2^ = 0.10882, *F* = 94. 874, *p* = 0.001) and between the ontogenetic stages (*R* ^2^ = 0.11093, *F* = 32.233, *p* = 0.001). A pairwise comparison corroborates these results, with all ontogenetic groups being significantly different to each other (*p* < 0.001, Table 1). No significant differences in shape were found between left and right sides of the jaws (*R* ^2^ = 0.00089, *F* = 0.6705, *p* = 0.567). Conversely, a small but significant difference in tooth shape (*R* ^2^ = 0.01551, *F* = 12.239, *p* = 0.001) and centroid size (*R* ^2^ = 0.00777, *F* = 6.9134, *p* = 0.001) between the palatoquadrate and Meckel's cartilage was detected with a Procrustes ANOVA, although an Analysis of Similarities (ANOSIM) revealed a significant overlap of teeth from upper and lower jaws (*R* = 0.044, *p* = 0.001). However, as the PCA results show, the factor describing most of the variation is the location of the teeth within the mesio‐distal axis of the jaws, which is also confirmed by a Procrustes ANOVA (*R* ^2^ = 0.49472, *F* = 62.5, *p* = 0.001).

**TABLE 1:** Results of the pairwise comparison to test for differences in tooth shape between the examined groups

Groups	d	UCL (95%)	Z	Pr > d
Embryo: Juveniles	0.05928876	0.02568046	7.550043	0.001*
Juveniles: Subadults	0.04657284	0.01743443	9.090736	0.001*
Subadults: Adults	0.05053828	0.01936540	8.467023	0.001*

Significance is depicted as *p*‐value (an asterisk indicates a *p*‐value <0.05). D, distance; UCL, upper confidence limit; Z, Z‐score.

### 3.4 Monognathic heterodonty

**FIGURE 10:**
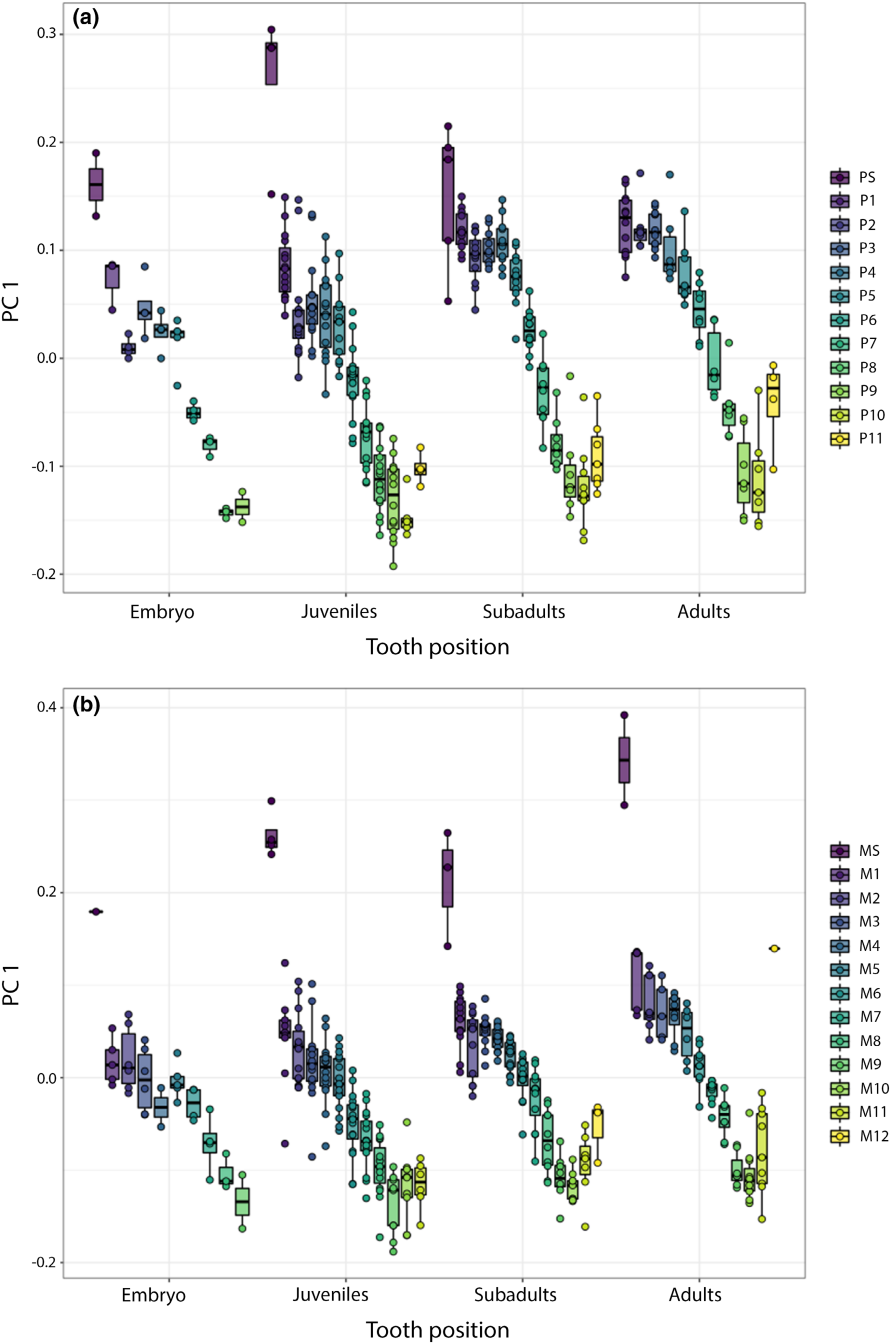
Relationship of tooth position and shape of the teeth in embryonic, juvenile, subadult and adult tiger sharks. (a) Palatoquadrate – (b) Meckel's cartilage

The changes in shape related to the position, as described by PC1 (Figure 9) were examined individually between each stage. Except for the symphyseal teeth, which are clearly different in shape to all others, a gradual shape change from teeth that are taller than wide in mesial positions to teeth that are wider than tall in distal positions is discernible. At position 10–11, a shift backwards occurs (Figure 10). In all ontogenetic groups, the position of the teeth along the mesio‐distal axis of the jaws explains more than half of the morphological variation (Table 2). Pairwise comparisons, however, revealed a gradual change of shape, with only the symphyseal teeth being different to the adjacent tooth file and all others showing no significant difference to the adjacent teeth (Table 3).

**TABLE 2:** Results of the Procrustes ANOVA to test for differences in tooth shape related to the position along the mesio‐distal axis on the jaws in the examined groups

Procrustes ANOVA							
Embryo	Df	SS	MS	Rsq	*F*	Z	Pr (<F)
Location	9	0.38269	0.042521	0.59313	9.8807	7.3783	0.001*
Residuals	61	0.26251	0.004303	0.40687			
Total	70	0.64519					

Juveniles	Df	SS	MS	Rsq	*F*	Z	Pr (<F)
Location	11	2.3402	0.212747	0.61395	40.048	12.217	0.001*
Residuals	277	1.4715	0.005312	0.38605			
Total	288	3.8117					

Subadults	Df	SS	MS	Rsq	*F*	Z	Pr (<F)
Location	12	1.7623	0.146855	0.61062	30.318	11.556	0.001*
Residuals	232	1.1238	0.004844	0.38938			
Total	244	2.8860					

Adults	Df	SS	MS	Rsq	*F*	Z	Pr (<F)
Location	11	1.63444	0.148585	0.62934	24.696	10.655	0.001*
Residuals	160	0.96265	0.006017	0.37066			
Total	171	2.59708					

Significance is depicted as *p*‐value (an asterisk indicates a *p*‐value <0.05).

**TABLE 3:** Results of the pairwise comparison to test for differences in tooth shape related to the position along the mesio‐distal axis on the jaws in the examined groups

Embryo	d	UCL (95%)	Z	Pr > d
S: 1	0.17595141	0.12244846	3.85089384	0.007*
1: 2	0.05676214	0.07743596	0.83352139	0.177
2: 3	0.02959267	0.07090839	−0.70372556	0.735
3: 4	0.03524801	0.08115653	−0.65207736	0.706
4: 5	0.02668509	0.07881246	−1.12868617	0.920
5: 6	0.05748304	0.07460156	0.89039875	0.163
6: 7	0.05038795	0.08181366	0.21478422	0.336
7: 8	0.05512897	0.08646827	0.26304112	0.346
8: 9	0.04905229	0.10179569	−0.41528642	0.595

Juveniles	d	UCL (95%)	Z	Pr > d
S: 1	0.21065182	0.08179635	8.33145185	0.001*
1: 2	0.03878299	0.05310109	0.79353422	0.199
2: 3	0.01938950	0.04827068	−0.71888994	0.746
3: 4	0.01878187	0.04823700	−0.77852426	0.776
4: 5	0.01815770	0.04959918	−0.83087914	0.804
5: 6	0.05083832	0.04996098	2.10232118	0.047*
6: 7	0.03900639	0.04664332	1.17729228	0.124
7: 8	0.04113690	0.05033426	1.19256919	0.122
8: 9	0.04947314	0.05353118	1.54375361	0.091
9: 10	0.02620282	0.06573920	−0.66832751	0.705
10: 11	0.07563368	0.07735284	1.76697429	0.059

Subadults	d	UCL (95%)	Z	Pr > d
S: 1	0.10413806	0.07136331	3.85646968	0.005*
1: 2	0.03527100	0.05093424	0.65156094	0.225
2: 3	0.01892013	0.05423532	−0.83969490	0.821
3: 4	0.01551996	0.05307886	−1.09489109	0.925
4: 5	0.03011291	0.05109306	0.15557761	0.363
5: 6	0.03848355	0.05013659	0.92949110	0.166
6: 7	0.03884882	0.03884882	0.87094441	0.179
7: 8	0.05213982	0.05705608	1.56957614	0.081
8: 9	0.05639065	0.06331205	1.47174085	0.093
9: 10	0.03491076	0.06565082	−0.05525344	0.423
10: 11	0.04452213	0.06174078	0.65733347	0.226
11: 12	0.07441220	0.11953719	0.38403941	0.281

Adults	d	UCL (95%)	Z	Pr > d
S: 1	0.24698768	0.15445953	4.43484834	0.003*
1: 2	0.02802981	0.07240042	−0.76296951	0.772
2: 3	0.01740898	0.07390507	−1.33174260	0.972
3: 4	0.02657666	0.07251666	−0.78481808	0.786
4: 5	0.02950131	0.07614750	−0.76085661	0.769
5: 6	0.04021619	0.07348090	−0.02371332	0.440
6: 7	0.04259334	0.06996955	0.15718365	0.357
7: 8	0.03824412	0.07840110	−0.20758970	0.500
8: 9	0.06400350	0.07578062	1.21932393	0.128
9: 10	0.04346535	0.07151264	0.21795371	0.342
10: 11	0.07567254	0.07591101	1.89116795	0.052

Significance is depicted as *p*‐value (an asterisk indicates a *p*‐value <0.05).

**FIGURE 11:**
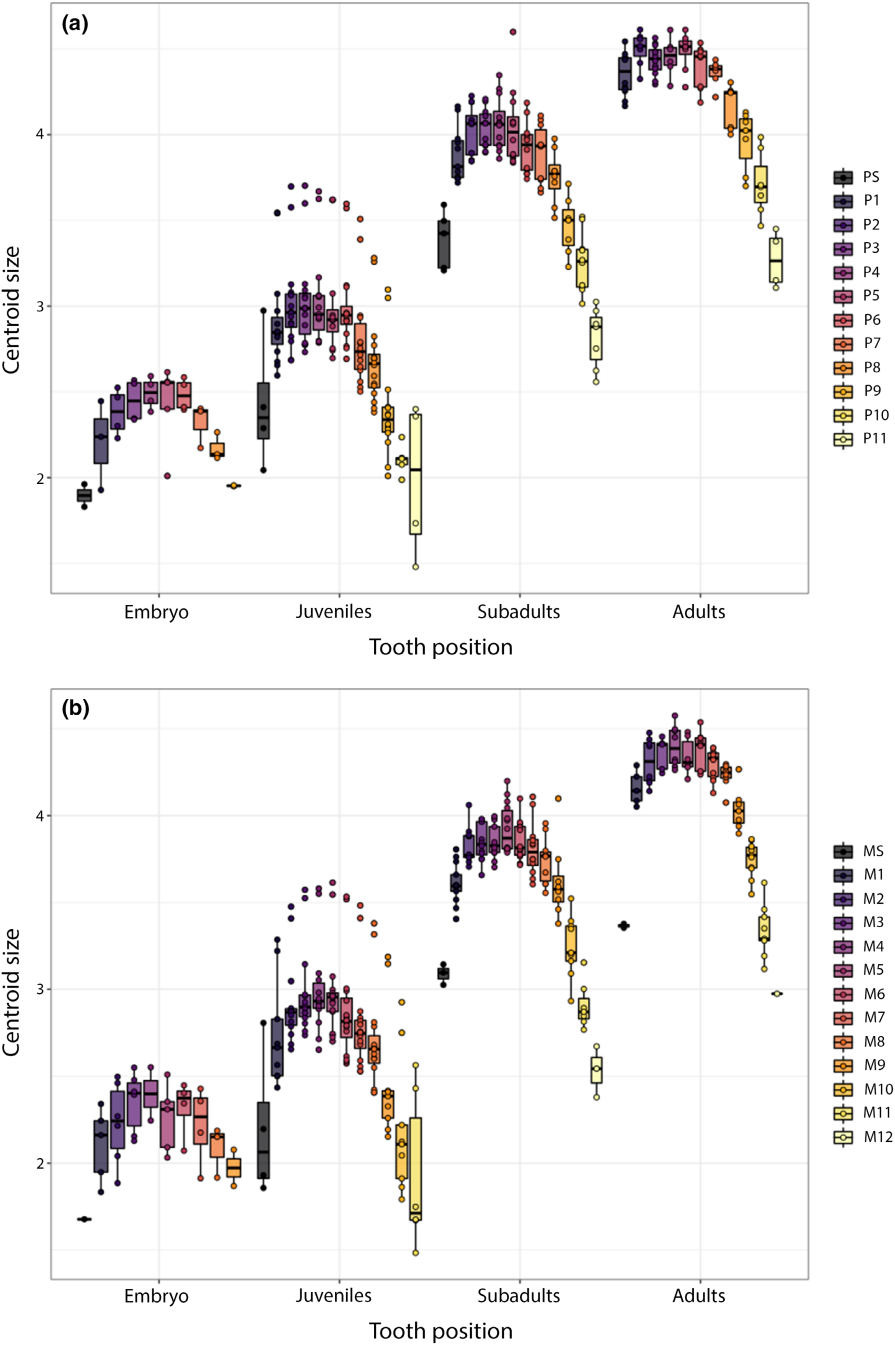
Relationship of tooth position and size of the teeth in embryonic, juvenile, subadult and adult tiger sharks. (a) Palatoquadrate – (b) Meckel's cartilage

To examine the differences in size (measured as log centroid size) within each tooth position and between each stage, we plotted the size values range along the mesio‐distal axis of the jaws. The plot depicts the size variation in all ontogenetic groups. First, a gradual increase in size is observable from symphyseal to more distal located teeth, with the largest teeth being those of positions three to five. The teeth then decrease in size and the distal‐most teeth (‘posteriors’) represent the smallest teeth in the jaws (Figure 11).

### 3.5 Ontogenetic trajectories

Given the morphological equality of teeth from both left and right sides of the jaws and the negligibly small difference between teeth from upper and lower jaws as shown above, teeth from all quadrants of the jaws were included in these analyses. However, only teeth that were available from all ontogenetic stages were included (i.e. teeth from files one to nine).

**FIGURE 12:**
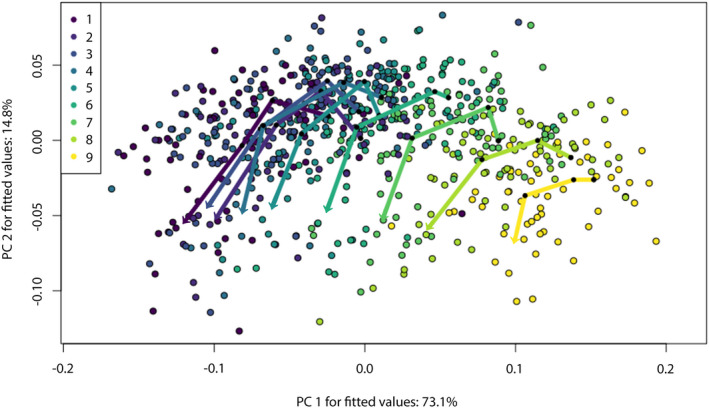
Tooth developmental trajectories of *Galeocerdo cuvier* for tooth files one to nine. Trajectories are drawn between the mean shape of the embryo (starting point), juveniles, subadults and adults (arrow tips)

**FIGURE 13:**
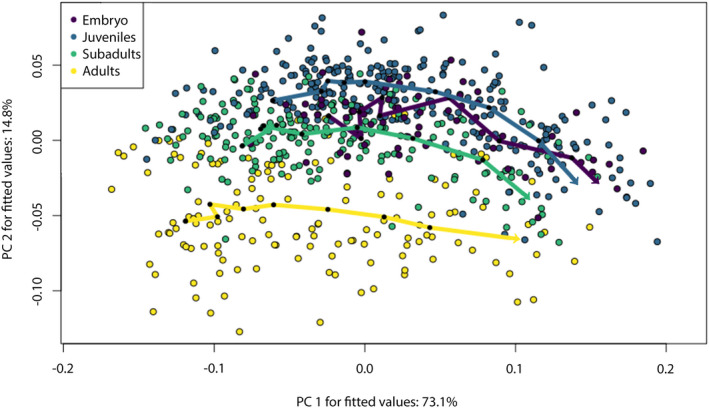
Tooth developmental trajectories of *Galeocerdo cuvier* for four ontogenetic stages. Trajectories are drawn along the mesio‐distal axis, from file one (starting point) to nine (arrow tips)

No significant differences were detected between the adjacent trajectory angles of tooth files one to nine, drawn between the subsequent ontogenetic stages from the embryo to adults (solid lines in Figure 12). However, slight differences in trajectory path distances were found between tooth files one to two (dΔ = 0.037, effect size Z = 2.143, *p* = 0.039) and two to three (dΔ = 0.035, effect size Z = 2.236, *p* = 0.029). The path distances between all others are not significantly different, as is the shape differences of all trajectories (Table 4). The comparison of the trajectories drawn from mesial to distal tooth files (one to nine) for each ontogenetic group (Figure 13) revealed significant differences in all trajectory angles (*p* < 0.05) but no differences in shape or path length (Table 5).

**TABLE 4:** Trajectory values between the adjacent tooth files, drawn between the subsequent ontogenetic stages (embryo to adult)

Correlations between trajectories					
	*r*	Angle	UCL (95%)	Z	Pr > angle
1: 2	0.9463228	0.3291336	0.4825478	0.3465170	0.310
2: 3	0.9705153	0.2434369	0.4648560	−0.4266556	0.611
3: 4	0.9596741	0.2849556	0.5130207	−0.2925293	0.557
4: 5	0.9314718	0.3723588	0.5201252	0.5041690	0.250
5: 6	0.9520880	0.3108041	0.4695178	0.1566867	0.366
6: 7	0.9491456	0.3202854	0.4943687	0.1284890	0.389
7: 8	0.9606551	0.2814450	0.5370098	−0.3600162	0.570
8: 9	0.8460097	0.5623401	0.6092674	1.2637778	0.076

Shape difference between trajectories				
	d	UCL (95%)	Z	Pr > d
1: 2	24.241.284	0.2581798	1.501038580	0.082
2: 3	24.455.371	0.2547897	1.525109276	0.074
3: 4	16.366.375	0.2654475	−0.032008376	0.472
4: 5	21.682.642	0.2661257	0.881705018	0.174
5: 6	23.070.330	0.2544124	1.289679340	0.105
6: 7	6.488.842	0.2700108	−1.771262071	0.981
7: 8	17.238.900	0.2729242	0.001566067	0.468
8: 9	29.243.972	0.3061003	1.544443554	0.069

Path distances between trajectories				
	d	UCL (95%)	Z	Pr > d
1: 2	373.782.749	0.03479183	2.14369090	0.039*
2: 3	359.871.749	0.03237212	2.23604747	0.029*
3: 4	192.144.946	0.03566188	0.42446587	0.289
4: 5	46.828.441	0.03620286	−0.89004244	0.792
5: 6	203.526.837	0.03211116	0.72127239	0.213
6: 7	29.903.539	0.03401908	−1.05946591	0.875
7: 8	56.993.502	0.03628188	−0.80075160	0.766
8: 9	85.772.595	0.04049436	−0.62976362	0.678

Significance is depicted as *p*‐value (an asterisk indicates a *p*‐value <0.05).

**TABLE 5:** Trajectory values between the ontogenetic stages, drawn from mesial to distal tooth files

Correlations between trajectories					
	*r*	Angle	UCL (95%)	Z	Pr > angle
Embryo: Juveniles	0.9823510	0.1881551	0.1842107	1.865795	0.041*
Juveniles: Subadults	0.9587522	0.2882170	0.1238312	8.905104	0.001*
Subadults: Adults	0.9801510	0.1995746	0.1387305	4.280924	0.002*

Shape difference between trajectories				
	d	UCL (95%)	Z	Pr > d
Embryo: Juveniles	2.172.635	0.3177824	−0.711437923	0.766
Juveniles: Subadults	1.773.506	0.2131068	0.395036050	0.323
Subadults: Adults	1.826.721	0.2461965	−0.366945332	0.622

Path distances between trajectories				
	d	UCL (95%)	Z	Pr > d
Embryo: Juveniles	0.0847733447	0.1312042	0.2015934	0.402
Juveniles: Subadults	0.0101200277	0.0450819	−0.5669581	0.645
Subadults: Adults	0.0008870641	0.0566289	−1.3787007	0.980

Significance is depicted as *p*‐value (an asterisk indicates a *p*‐value <0.05).

## 4 DISCUSSION

### 4.1 Dignathic and monognathic heterodonty

The here presented morphometric analyses confirm previous qualitative observations on the heterodonty of tiger sharks (Compagno, 1988). The dignathic heterodonty has been described to be fairly weak and that teeth from upper and lower jaws barely differ from each other morphologically, but in size (upper teeth are slightly larger than the lower ones; Snodgrass & Heller, 1905; Sarangdhar, 1943; Hooijer, 1954). This is in accordance with our quantitative analyses that pointed out differences in shape and size between teeth from the palatoquadrate and the Meckel's cartilage, although their morphospaces are highly overlapping, exposing that these differences are rather miniscule. Carcharhiniform sharks typically exhibit a quite distinct dignathic heterodonty, that is with broad‐crowned teeth in the palatoquadrate and narrow teeth in the Meckel's cartilage (e.g. Cullen & Marshall, 2019). Tiger sharks form together with Carcharhinidae and Sphyrnidae a highly derived clade within the carcharhiniform sharks (López et al., 2006; Naylor et al., 2012) and, thus, the weak dignathic heterodonty exhibited by *Galeocerdo cuvier* appears to be a derived rather than a primitive character. The oldest fossil representative of the tiger sharks, †*Galeocerdo eaglesomei* White 1955, presumably still exhibited a distinct dignathic heterodonty (Samonds et al., 2019), while younger tiger shark species reduced the degree of heterodonty (Türtscher et al., 2021).

The monognathic heterodonty in *Galeocerdo cuvier* follows the same pattern in all studied ontogentic stages, characterized by a gradual shape and size change from mesial to distal tooth positions. A similar gradual monognathic heterodonty can also be observed in other carcharhiniform sharks (e.g. Berio et al., 2020; Cullen & Marshall, 2019). In several lamniform sharks (the sister group to carcharhiniform sharks), however, the monognathic heterodonty is much more distinct and it is possible to easily distinguish between anterior, intermediate and lateral teeth based on the tooth morphology (Shimada, 2002a). This might be attributed to the dental bulla which is characteristic for lamniform sharks, a feature that tiger sharks and carcharhiniform sharks in general, with the exception of the snaggletooth shark *Hemipristis elongata* (Klunzinger, 1871), do not possess.

The weakly pronounced monognathic and dignathic heterodonty in tiger sharks has probably evolved as a specialization to facilitate the shark's ability to capture and handle large prey. Together with the characteristic cockscomb‐shaped tooth morphology and the lateral side‐to‐side movement of the head (Clua et al., 2013; Randall, 1992), the tiger shark dentition functions like a saw and, therefore, allows it to easily cut through even the largest and hardest prey (e.g. sea turtles).

### 4.2 Ontogenetic tooth morphology

Tiger sharks are reportedly known to exhibit an ontogenetic dietary shift, with juvenile tiger sharks feeding on smaller prey like teleosts and cephalopods, while adult specimens feed on larger prey such as sea turtles, mammals and other elasmobranchs (e.g. Dicken et al., 2017; Lowe et al., 1996). Fu et al. (2016) investigated ontogenetic changes of the caudal fin and head shape in tiger sharks and found a shift from conical to broad and blunt head shapes with increased size and age. They assumed a correlation between a blunt head enabling a larger attachment area for muscles (Huber et al., 2009) and the possibility for the sharks to prey on larger and harder prey items (e.g. sea turtles). However, they did not include tooth shapes in their study but indicated the necessity for pending research on ontogenetic tooth morphology and the potential link to shifting diets in tiger sharks (Fu et al., 2016). Our results highlight weak dental heterodonties between the examined ontogenetic groups, characterized by a gradual and subtle shape change with increasing size being the most important factor. These results indicate that ontogenetic dietary variations might not be dependent on tooth morphology. However, we found a subsequent increase in tooth complexity and number of serrations from embryonic to adult specimens. Also, the histological differentiation of complex serrations into primary and secondary serrations happens rather late, with juvenile specimens only exhibiting secondary serrations on the mesial cutting edge (albeit an increase in complexity is already seen morphologically), while complex serrations in adults are distinctly developed and histologically distinguishable on all cutting edges. According to Moyer and Bemis (2017), the more complex the teeth are, the lower is the pressure per serration and the more effective are the serrated edges, suggesting that this might assist in preying on larger and harder prey. They further hypothesize that secondary serrations might also function as stress concentration points that reduce the rate of wear of the primary serrations so that the teeth remain fully functioning and sharp for as long as possible (Moyer & Bemis, 2017). It is worth noting that extinct tiger shark species with presumably large body sizes, for example †*G. capellini* Lawley 1876 and †*G. mayumbensis* Dartevelle and Casier 1943, also had double serrated teeth, whereas smaller species, for example †*G. aduncus* (Agassiz, 1835) exhibited teeth with simple serrations (Türtscher et al., 2021). This insight in the evolutionary history of tiger sharks indicates that tooth complexity might facilitate larger body sizes, however, further mechanical studies are needed to fully understand the impact of secondary serrations.

The median number of tooth files observed in all ontogenetic groups of tiger sharks was 11 for both sides of upper and lower jaws. In our sample, the lowest number of tooth files is 10, while the highest is 12. Previously, Compagno (1988) described a possible number of up to 13 tooth files in a jaw, while Lowry et al. (2009) included a specimen with only nine tooth files in one jaw in their study. Our results nevertheless solidly confirm that the number of tooth files is very constant throughout the ontogeny in tiger sharks. Similar observations in tiger sharks were reported by Reif (1984), as distinguished from other species that experience an increase in tooth file numbers over time, for example bullhead sharks *Heterodontus* (Reif, 1976), porbeagle sharks *Lamna nasus* (Bonnaterre, 1788) (Purdy & Francis, 2007), white sharks *Carcharodon carcharias* (Tomita et al., 2017) or nursehounds *Scyliorhinus stellaris* (Linnaeus, 1758) (Berio et al., 2020).

The amount of teeth per tooth file gradually increases in tiger sharks with ontogeny, from four in embryonic to up to seven in adult specimens. A higher number of teeth per tooth file might be linked with a faster tooth replacement (Moyer et al., 2015). However, tooth replacement rates are impacted by various factors, including tooth imbrication patterns or water temperature (Correia, 1999; Luer et al., 1990; Strasburg, 1963).

### 4.3 Embryonic dentition

The examined embryo in the present study had a total length of 56 cm and therefore was presumably close to birth, as tiger sharks measure between 51 cm (Compagno, 1984) and 90 cm (Simpfendorfer, 1992; Whitney & Crow, 2007) total length at birth. The oldest (labial‐most positioned) teeth of the embryo were the smallest ones. They were less mineralized than the subsequent teeth of the tooth file and did not yet display the typical, distinct tiger shark tooth morphology, nor any serrations. Younger teeth, on the other hand, were more mineralized and markedly larger, indicating rapid growth of the embryo. They already had the typical tooth morphology of juvenile tiger sharks, with noticeable simple serrations. A marked increase in size from labially to lingually positioned teeth was also reported in embryos of other species, for example the sand tiger shark *Carcharias taurus* Rafinesque, 1810 (Gomes & dos Reis, 1990), the crocodile shark *Pseudocarcharias kamoharai* (Matsubara, 1936) (Cigala‐Fulgosi, 1992), the porbeagle shark *Lamna nasus* (Purdy & Francis, 2007) and the white shark *Carcharodon carcharias* (Tomita et al., 2017).

None of the embryo's teeth were in an erect position and it, therefore, did not exhibit a functional dentition. In contrast, a previous study reported peg‐like teeth in an erect position in two tiger shark embryos measuring 55 cm TL (Reif, 1984). These teeth showed the embryonic tooth morphology and were already shed in some files. However, due to the rudimentary morphology of the erect teeth and the low degree of mineralization that we observed in our embryo in the labial‐most tooth row, we suppose that the described erect teeth by Reif (1984) do not constitute true functional teeth. Rather, they are an artefact of tooth progression due to the morphological transition towards a juvenile tooth morphology. This assumption is further supported by previous studies on shark tooth development, which defined functional teeth to be both in an erect position and fully mineralized (Jambura et al., 2018; Moyer et al., 2015; Schnetz et al., 2016). In the present study, we observed no erect teeth in the embryonic specimen, however, shed and swallowed teeth with embryonic tooth morphology were recovered from the stomach and intestine (Figure 8), revealing that tooth replacement was already in progress in the specimen.

A functional embryonic dentition is known from several lamniform sharks, including the white shark *Carcharodon carcharias* (Tomita et al., 2017); white sharks already develop functional teeth early in their embryonic development, but these are morphologically different from those of older embryos and specimens after birth. This functional, peg‐like dentition develops concomitant with the oophagous phase in which the early‐term embryos actively feed on nutritive eggs provided by the mother. Mid‐term embryos are nourished by the accumulated eggs in the stomach and consequently lose the functional teeth, which are swallowed and subsequently replaced with an ‘adult‐type’ dentition. Late embryos close to birth again exhibit functional, ‘adult‐type’ teeth (Tomita et al., 2017). Other lamniform sharks with documented functional embryonic dentitions include the sand tiger shark *Carcharias taurus* (Shimada, 2002b), which reportedly develop functional, peg‐like teeth very early during development to free themselves from the egg cases and to feed subsequently on their younger siblings (Naidoo et al., 2017). The specialized nourishment during the embryonic development allows both *C. carcharias* and *C. taurus* to have large pups but relatively small litter sizes (Ebert et al., 2013), in contrast to tiger sharks with smaller pups but large litter sizes between six and 82 young per litter (mean number of around 30) (Bigelow & Schroeder, 1948; Simpfendorfer, 1992). Contrary to lamniform sharks that already search and process food actively in utero, tiger shark embryos do not depend on functional teeth: during gestation, tiger sharks provide their offspring with yolk and nutritive fluid (i.e. embryotrophe) that is imbibed by the embryos (Castro et al., 2016).

We hypothesize that the embryo examined here as well as those studied by Reif (1984) show the transition from an embryonic to a juvenile tooth morphology, and, therefore, that the dental morphological shift and the concomitant creation of a true functional tooth row happens either very late prenatal or very early postnatal.

### 4.4 Tooth file reversal

Reversed tooth files comprise teeth that are mirrored compared to the other teeth of the jaws resulting in that the apex is directed mesially instead of distally. Such reversed files were documented in sharks before. Antunes (1963), for example reported one symmetrical and three reversed tooth files in a tiger shark palatoquadrate. Later, Compagno (1967) reported on three more shark species with similar observations: a reversed tooth file was observed in one specimen each of the smooth hammerhead *Sphyrna zygaena* (Linnaeus, 1758), the tope *Galeorhinus galeus* (Linnaeus, 1758) and the pacific sleeper shark *Somniosus pacificus* Bigelow & Schroeder, 1944, with the affected teeth having also slightly different morphologies compared to the adjacent normally orientated teeth. Gomes and dos Reis (1990) even found a reversed tooth file in an embryonic sand tiger shark *Carcharias taurus*. Other shark species with documented reversed tooth files include the whitetip reef shark *Triaenodon obesus* (Rüppell, 1837), and the leopard shark *Triakis semifasciata* Girard, 1855 (Reif, 1980), the night shark *Carcharhinus signatus* (Poey, 1868) (Raschi et al., 1982), the gulper shark *Centrophorus granulosus* (Bloch & Schneider, 1801) (Smith et al., 2013), the basking shark *Cetorhinus maximus* (Gunnerus, 1765) (Welton, 2013), the nursehound *Scyliorhinus stellaris* (Berio, 2021), the sharptooth lemon shark *Negaprion acutidens* (Rüppell, 1837) (R. Kindlimann, pers. obs.), the blue shark *Prionace glauca* (Linnaeus, 1758), the scalloped hammerhead *Sphyrna lewini* (Griffith & Smith, 1834) (J. Türtscher, pers. obs.) and the bull shark *Carcharhinus leucas* (Müller & Henle, 1841) (P. L. Jambura, pers. obs.). In white sharks *Carcharodon carcharias*, the third tooth in the palatoquadrate (intermediate tooth, ‘eye tooth’) is typically reversed in polarity (Applegate & Espinosa‐Arrubarrena, 1996; Hubbell, 1996; Ehret et al., 2012). This pattern is unique among lamniform sharks and is already established in embryonic white sharks (Tomita et al., 2017). However, the reversed polarity observed in white sharks represents a consistent species‐specific pattern and, therefore, cannot be compared with the random reversals documented in other shark species.

In the present study, six tiger shark jaws with one reversed tooth file each were recognized. No identifiable underlying cause of this discrepancy, such as stingray or teleost spines embedded in the jaws (e.g. Andre, 1784; Becker et al., 2000; Gudger, 1937) could be detected. However, a striking detail of this dental aberration is that the mirrored tooth file polarity in tiger sharks seems to affect distally positioned tooth files: taking into account the present study and the study of Antunes (1963), a total of seven (77.77%) files in distal‐most positions were affected, only two corresponding files (22.22%) were located further mesial (LP10 in Antunes (1963) and LP8 in the present study). It is possible that distal tooth files are more prone to tooth aberrations than mesial ones, however, most likely this conspicuity represents a coincidence. Reif (1980) introduced a ‘polarity switch model’, in which a morphogen gradient in the tooth germ determines the ultimate morphology of the teeth within the tooth file. Depending on the concentration of the morphogen, possibly affected by an injury of the dental lamina or a discrepancy in a regulatory process, the morphology or polarity of the resulting teeth might differ from others (Reif, 1980). According to this model, the fate of each tooth file depends on its own, independent morphogen gradient, which would make each tooth file equally prone to polarity reversals. The increased incidence of tooth file reversals at distal positions in tiger sharks would be better explained if the morphogen gradient extended over the entire jaw ramus. However, more studies on this topic are needed for eventual clarification. Also, further investigation on tooth polarity, preferably across several species, is necessary to understand the recurrent phenotypes we describe.

## 5 CONCLUSIONS AND PERSPECTIVES

Until now, dental morphological studies on tiger shark teeth mostly focused on extinct species (see Türtscher et al., 2021). Here, we provide the first comprehensive quantitative study on intraspecific tooth variations in the extant tiger shark *Galeocerdo cuvier* based on four developmental stages ranging from late embryo to adults. Our results confirm a weak ontogenetic heterodonty in tiger sharks, characterized by gradual ontogenetic changes in tooth morphology from juveniles to adults. Also, a conserved tooth file count over time and a successive increase of replacement teeth is revealed. Distinct shape variations along the mesio‐distal axis are present in all stages, with mesial‐most and distal‐most positions exhibiting the most variances. Additionally, to our best knowledge, we provide the first detailed description of embryonic dental morphologies in tiger sharks here. The results of this study significantly augment our understanding of tiger shark life history; we provide new insights into prenatal dental traits and thus into the unique reproduction mode and nutrition strategy in utero, as well as knowledge about possible form‐function relationships of the teeth associated with the ontogenetic stage of the animals. Similarly, the fossil history of tiger sharks indicates that more complex teeth facilitated larger body sizes. This study also contributes to our understanding of tiger sharks in deep time, since it is only possible to draw conclusions about fossil species if we understand the extent of heterodonty in living sharks.

More studies on tooth morphologies including embryos and juveniles are necessary to thoroughly describe the early tooth development in elasmobranchs and to establish the early dental morphology, which might differ from that of adults. Such information is important as dental traits often represent the only available information of extinct species. This therefore will aid in correctly identifying ambiguous fossil taxa (especially those that are based on micro‐teeth) and therefore augment our understanding of elasmobranch communities and their ecology as well as diversity patterns in deep time.

## CONFLICT OF INTEREST

None declared.

## Supporting information


Table S1

Table S2

## Data Availability

The data that support the findings of this study are available from the corresponding author upon reasonable request.
